# Premobilization of CD133+ cells by granulocyte colony- stimulating factor attenuates ischemic acute kidney injury induced by cardiopulmonary bypass

**DOI:** 10.1038/s41598-019-38953-5

**Published:** 2019-02-21

**Authors:** Xiaoqiang Li, Qin Wan, Jie Min, Linjia Duan, Jin Liu

**Affiliations:** 1 Department of Anesthesiology, West China Hospital, Sichuan University, 610041 Chengdu, China; 2 Department of Integrated Traditional and Western Medicine, West China Hospital, Sichuan University, 610041 Chengdu, China; 3 Department of Cardiology, West China Hospital, Sichuan University, 610041 Chengdu, China

## Abstract

Ischemic acute kidney injury (IAKI) is a common but severe complication after a cardiopulmonary bypass (CPB). Multiple studies have demonstrated that peripheral CD133+ or differentiated cells are able to home and repair the damaged tissues, but the number of available CD133+ cells is limited, and no efficient method published previously to mobilize them immediately. We analyzed the relationship between CD133+ cells and renal function in CPB patients, in addition, the efficacy of granulocyte colony-stimulating factor (G-CSF) pre-mobilized CD133+ cells in treating of mouse IAKI model have been investigated. In the clinical study, the prospective cohort study analyzed the correlation between BUN/Crea level and the peripheral CD133+ cell numbers. CPB was associated with postoperative renal dysfunction. The significant negative correlation was observed between patients’ Crea and CD133+ cells (P < 0.05). The proposed mechanism studies were performed on the mouse IAKI model. The experimental mice were treated by G-CSF to mobilize CD133+ cells before implementing CPB. Data on cell count, inflammatory index, renal function/injury, and CD133+ cell mobilization were analyzed. The result demonstrated that pretreatment by G-CSF resulted in tremendous increase in the number of mouse peripheral blood and renal CD133+ cells, significantly reduces renal tissue inflammation and dramatically improves the renal function after CPB. In summary, we concluded that premobilization of CD133+ cells abated CPB induced IAKI, by promoting both repairing damaged epithelium and by its anti-inflammatory activity. Our findings stress the remarkable applications of CD133+ or differentiated cells-based therapies for potential preventing ischemic acute kidney injury.

## Introduction

Ischemic acute kidney injury (IAKI) is a severe complication which occurs in about 30% of patients after implementing the cardiopulmonary bypass (CPB), and 2–7% of reported patients might need renal replacement therapy and associated with 50% mortality^1,2^. It is one of the subtypes of acute kidney injury (AKI), a complex diagnosis, caused by ischemia and/or ischemia/reperfusion injury in kidney^3–10^. In addition, 30–70% of the patients who survived their IAKI still have a high risk of developing or exacerbating chronic kidney diseases and hastened the development of end-stage renal disease^1^. However, IAKI can be early diagnosed by monitoring the rapidly decreased kidney function which indicated by an elevated serum creatinine level. Therefore, it can be a highly effective way of staving off the incidence If handled properly.

Prevention and select a proper therapy plan of IAKI still remains a challenge, and currently, there are few ways to achieve it efficiently. However, several studies showing the relatively high efficiency of CD133+ cell-based therapies for cardiovascular disease, limb ischemia, stroke, diabetic wounds and acute lung injury^11–15^ suggest the possibility of using CD133+ cells to treat IAKI since all of the above-mentioned diseases share similar causes with IAKI- ischemia. CD133+ cells are a class of ‘stem/progenitor’ like cells comprising a plurality of subsets, with self-renewal, high proliferation, and multilineage differentiation capabilities^16^. CD133+ cells have a wide range of functions such as promoting angiogenesis, mediating tissue regeneration and regulating inflammation^17–22^. Missol-Kolka *et al*. reported that CD133+ cells can be detected in both human and rodent prostate luminal cells, indicating that CD133 may not be exclusively expressed in the basal stem cells^23^. More interestingly, Bussolati’s and Ikehara’s group reported that in the drug-induced mouse AKI model, exogenous CD133+ cells have the ability, promoting renal cell proliferation and survival, regulating inflammation, reducing renal tubular necrosis, thereby improving renal function and reducing kidney damage^24,25^. However, the source of homing CD133+ cells has existed in circulation^26–28^ which limited the efficiency of therapeutic applications in human studies. Studies have shown that continuous subcutaneous injection of some cytokines, such as Granulocyte colony-stimulating factor (G-CSF) can increase the number of CD133+ cells in circulation up to ten times^29,30^, a process termed “mobilization.” The increase of the number of cells in circulation by this above-mentioned process enlarged the source of homing cells, therefore potentially could exaggerate the potency of cell-based therapy. Thus, we hypothesized that mobilization of CD133+ cells has the capacity to improve its clinical efficacy against CPB-induced injury, especially in IAKI.

Granulocyte colony-stimulating factor (G-CSF), also known as colony-stimulating factor 3 (CSF-3), is a glycoprotein that stimulates the bone marrow to produce granulocytes and stem cells and release them into the bloodstream^31,32^. The pharmaceutical analogs of naturally occurring G-CSF are called filgrastim and lenograstim, works well for mobilizing progenitor cells in patients undergoing bone marrow transplantation^33–35^. Other studies have shown that exogenous G-CSF not only increases the number of CD133+ cells in all circulating stem cells populations, also accelerate the improvement in renal functions and prevent the chemotherapy induced renal injury^24,36–38^. In fact, endogenous level of G-CSF rapidly increases in patients after cardiac surgery including CPB, suggesting that it may serve as a natural mobilizing agent for self-repairing by the body^33–35,39,40^. But a notable disadvantage in relying on endogenous G-CSF is that it takes at least 24 hours to mobilize CD133+ cells, based on various experimental and clinical studies^41,42^. However, IAKI often occurs within 24–48 hours after CPB surgery^43^.

In this project, we tested the relationship with CD133+ cells and CPB induced postoperative renal dysfunction in patients, in addition, we test the efficacy of mobilization of cells by G-CSF in treating of mouse IAKI model. Finally, we investigated a strategy to abated cardiopulmonary bypass-induced ischemic acute kidney injury by using premobilized CD133+ cells, which effectively reduces the CPB induced IAKI and improves renal function.

## Materials and Methods

### Patient characteristic

The clinical part’s study used in this manuscript approved by the Sichuan University Ethics Committee and registered with the China Clinical Laboratory Registration Center (No. ChiCTR- OCS-09000398; www.chictr.org/). Research comply with the Code of Ethics of the World Medical Association (Declaration of Helsinki).

The clinical part’s study was designed in a prospective cohort fashion. A total of 138 patients with CPB were enrolled, related data was conducted in 138 adult patients and time interval was between April 2015 and June 2016 in Western China Hospital. All patients’ procedure was established based on the primary disease (include but not limit Coronary Artery Bypass mitral valve replacement aortic valve replacement and double valves replacement), The history, design, and characteristics of studies have listed as following the inclusion criteria: (1) hemodynamic abnormalities; (2) Age between 20–60 years old. And exclusion criteria: (1) unwilling to provide consent for the study; (2) coagulation dysfunction (INR > 1.3), low peripheral blood platelet count (<50 × 10^9^/L) or anti-coagulation drugs usage; (3) any types of basic renal diseases; (4) pre-operative coexistent diseases, such as acute cholangitis acute pancreatitis, digestive tract hemorrhage, severe liver disease, and septic shock; (6) pregnant women. Written informed consent was obtained from each patient him/herself or their authorized family members. The patients were treated with adequate antibiotics to prevent infection.

### Experimental Animals

C57BL/6 mice were purchased from the Animal Experiment Centre of Sichuan, China. All mice were housed in Sichuan University’s Biological Resources Unit in accordance with guidelines of the Association for Assessment and Accreditation of Laboratory Animal Care International. The experimental animal protocols were approved by the Institutional Animal Care and Use Committee of the Sichuan University. Mice were used at 12–20 wk of age in all experiments.

### IAKI Mouse Model

To induce IAKI model, mice were anesthetized, the respiratory rate was set at 130 breaths/min, and the tidal volume was 0.5 ml of room air. After administration of heparin (500 U/kg), one 24-gauge intravenous catheter (Becton Dickinson Medical Devices, USA) was inserted into the right carotid artery and another into the external jugular vein, and the two catheters were connected with a tube (inner diameter, 1/32 inch) primed with 0.4 ml normal saline. A roller pump (Stock II, Munich, Germany) was used to transfer blood from the artery to the vein at 5 ml/min for 30 minutes, thus attaining a mouse model of CPB’s extracorporeal circulation. In this system, significant kidney injury would be observed during the subsequent 60 minutes observation period.

### Determination of the number or the percentage of CD133+ cells in peripheral blood

The flow cytometry method is employed to analysis the number of CD133+ cells in human and mouse peripheral blood. After the RBC lysis processing, the blood cells were resuspended into the original volume into PBS, 1 μg/mL APC-anti-CD133 (anti-human-CD133, Miltenyi Biotec, USA; anti-mouse CD133, Bioss Antibodies, China. respectively) or same dose of isotype control was added, 30 mins later, expression levels of CD133 on the peripheral blood cells were measured by flow cytometry (BD Biosciences, USA).

### The expression level of the CD133+ cells in mouse kidney

The percentage of CD133+ cells in mouse renal tissue is expressed by the proportion of CD133+ cells that occupy the total number of nucleated cells in the renal tissue. The right kidney of the mice was obtained at the indicated time point of the experiment, and the percentage of CD133+ cells occupying the total renal cells in each group was determined. The experimental procedure is as follows: The kidneys collected from each group were washed with 10% heparin in 1x PBS to remove the blood, then were cut into fragments in 1x PBS at 4 °C. Then hyaluronidase (1 mg/ml), DNA lyase (1 mg/ml), collagenase I (150 units/ml) (Solarbio, Beijing, China) were added and the samples were kept at 37 °C in water bath for 1 hour with a gentle vertex in every 15 mins. The undigested tissue was filtered out by using 70 µm nylon cell filter (ThermoFisher, USA). The flow-through mixture was centrifuged at 600 g for 10 min at 4 °C, and the supernatant was removed. The cells were washed again by 1x PBS, and then the purified Rat anti-mouse CD16/CD32 (eBioscience, USA) were added as Fc blocking purposes. Followed by addition 30 minutes of anti-mouse CD133 staining (Biolegend, USA) and analyzed by flow cytometry (BD Biosciences, USA). Each sample counted at least 1000000 cells.

### Inflammatory factors Detection

Kidney Samples collected from each group of mice were diluted and used in respective ELISA to measure levels of Tumor Necrosis Factor-a (TNF-a) (ThermoFisher, USA) and Neutrophil Elastase (NE) (USCNLIFE, China) following manufacturer-provided protocols.

### Determination of renal function

The determination of the levels of nitrogenous end products of metabolism, creatinine (Crea) and blood urea nitrogen (BUN), were examed and analyzed by the Department of Laboratory Medicine, West China Hospital of Sichuan University by following AACC publishes Laboratory Medicine Practice Guidelines.

### Histology and Histo-immunofluorescence

Following sacrifice, the experiential mice, left kidneys were removed and fixed in 4% (vol/vol) Paraformaldehyde overnight, then embedded in OCT compound. Sections were cut at 4 μm on a microtome and stained with hematoxylin and eosin (H&E) to assess the renal injury or immunofluorescence to assess injured areas, by following established protocols. At the indicated time point of the experiment, the left kidney was used for pathological analysis. The pathological injury score of all the samples was assessed twice in a blinded fashion by different investigators, and the average score from the two assessments was recorded as the composite score. The grading criteria for renal injury were as follows: edema of tubular epithelial cells, loss of normal morphology, cytoplasmic vacuoles. The grading standard for renal tubular involvement is as follows: observing the above-mentioned characteristics of the samples under the same, 40x magnification microscope, Clinical severity was assessed with 0–5 scoring system. (0, no renal tubular involvement; 1, less than 25% renal tubular involvement; 2, 25–50% renal tubular involvement; 3, 50–75% renal tubular involvement; 4, more than 75% renal tubular involvement). Using the same method, a cumulative observation of 5 randomly selected cases of renal tubular involvement in the field of vision were scored, and the average value was used as the score for pathological renal injury.

### Statistical Analysis

All statistical analyses were performed with SPSS 24.0 statistical software. Continuous data were reported as the mean ± SD for parametric data and the median with interquartile ranges (IQR) for nonparametric data or as counts and percentages for categorical variables. Continuous variables were also expressed as ranges. The proportion of cases that accepted the examination for each aspect and the exclusive distribution of different pathological findings were described. A bivariate logistic regression model was established, univariate regression analysis was used to define significant relations between the CD133+ cells variables of Crea and/or BUN. p < 0.05 was considered statistically significant.

## Results

### Patient characteristics

The clinical study comprises 138 patients have CPB during the surgery (male: female = 37:101), during April 2015 and June 2016. Table 1 outlines the demographic and clinical characteristic variables for all enrolled cases. The mean age was 47.0 ± 9.3 years, ranged from 37 to 56.3, The mean arterial pressure(MAP) was 65.5 ± 10.6 mmHg, ranged from 49 to 80 mmHg. The mean nasopharyngeal temperature was 34.4 ± 0.5 °C, ranged from 33.5 to 35.1 °C. The mean urine output per hour CPB period was 69.8 ± 18.9 ml, ranged from 45 to 100 ml. Average CPB time is 120 min (55 min–334 min), average operation time is 3.83 hrs (2.25 hrs–9.50 hrs).

**Table 1:** Demographic and clinical characteristics of CPB patients and the outcome of the studied subjects.

Variable	Measure	Range
Gender (male/female)	37/101	Not available
Age	47.0 ± 9.3	37–56.3
Mean arterial pressure (MAP)	65.5 ± 10.6 mmHg	49–80 mmHg
Nasopharyngeal temperature	34.4 ± 0.5 °C	33.5–35.1 °C
Urine output per hour	69.8 ± 18.9 ml	45–100 ml
Average CPB time	120 min	55 min–334 min
Average operation time	3.83 hrs	2.25 hrs–9.50 hrs

### The patients Crea and BUN level have elevated after CPB 4 hours, and have a negative correlation with CD133+ cell numbers

**Figure 1:**
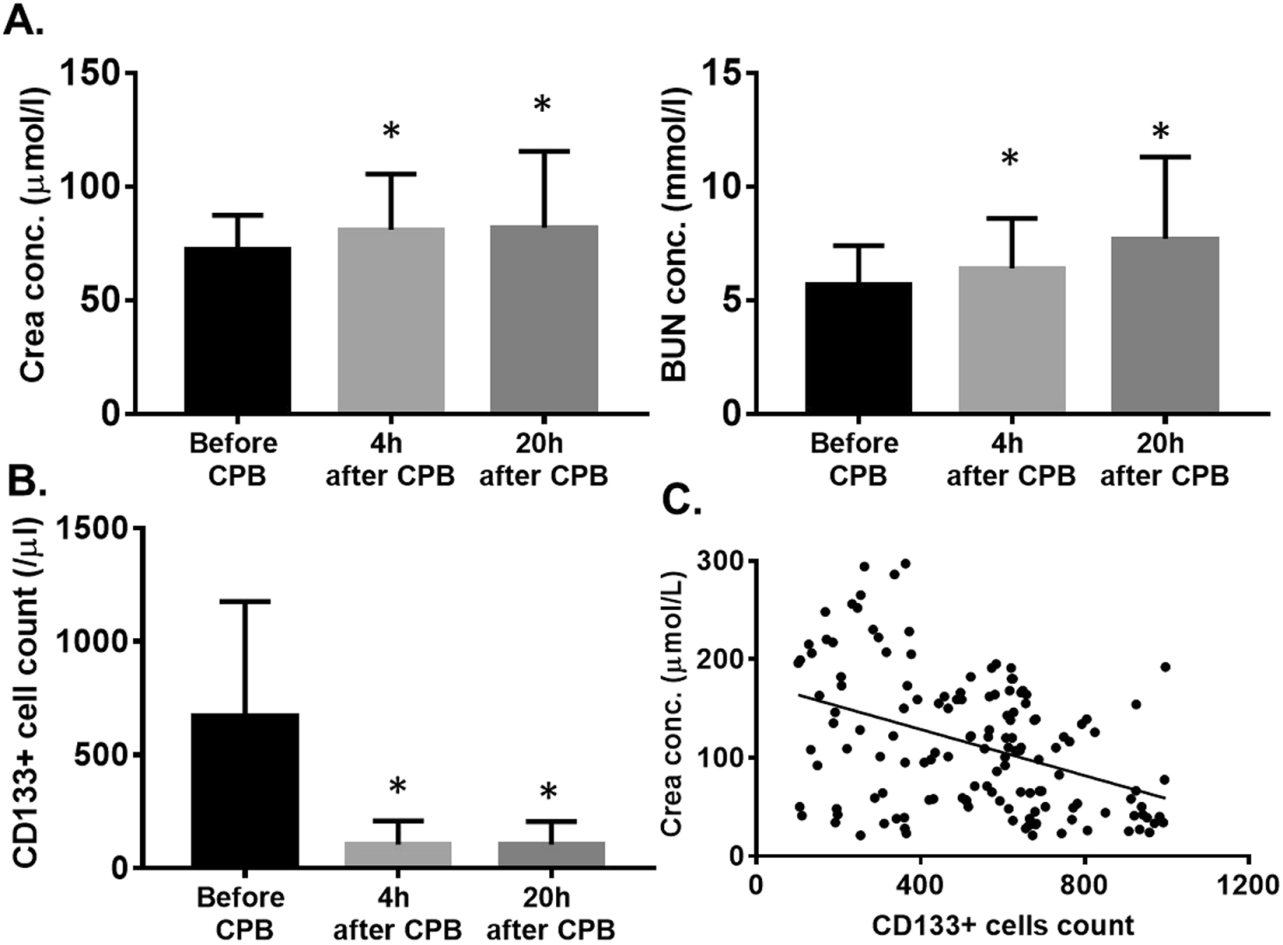
(**A**) The Serum Crea and BUN level have significantly elevated after CPB 4 hours and keep in the high concentration to the 20th hour; (**B**) Peripheral blood CD133+ cells count; (**C**) The Crea concentration has negative relevant with CD133+ cells. *Compared with the before CPB group, p < 0.05. (N = 138).

Our experiments are in line with previous results that CPB is associated with postoperative renal dysfunction. Compare to the serum samples collect before the CPB, the patients have transient renal dysfunction, which leads to the elevated serum Crea level after CPB 4 hours and keeps in the high concentration to the 20th hour. (72.6 ± 14.8 vs. 81.1 ± 24.5 vs. 81.9 ± 33.6 mmol/L), and the BUN level has the same significant increase trend (5.7 ± 1.7 vs. 6.4 ± 2.2 vs. 7.7 ± 3.6 mmol/L) (Fig. 1A,B). Furthermore, the peripheral blood CD133+ cells were isolated and analyzed by Flow cytometry, compare to the pre-operation condition, the peripheral blood CD133+ peripheral blood cell count showed significant decreased, (670 ± 506 vs. 104 ± 104 vs. 104 ± 101 cells/µl peripheral blood nucleated cells) (Fig. 1B), a significant negative correlation was observed between Crea and CD133+ cells (r = −0.1458; P < 0.05) (Fig. 1C), however, we haven’t any correlation between BUN and CD133+ peripheral blood cells (P > 0.05).

### The number of CD133+ cells in renal tissue increased in G-CSF pre-treatment group

**Figure 2:**
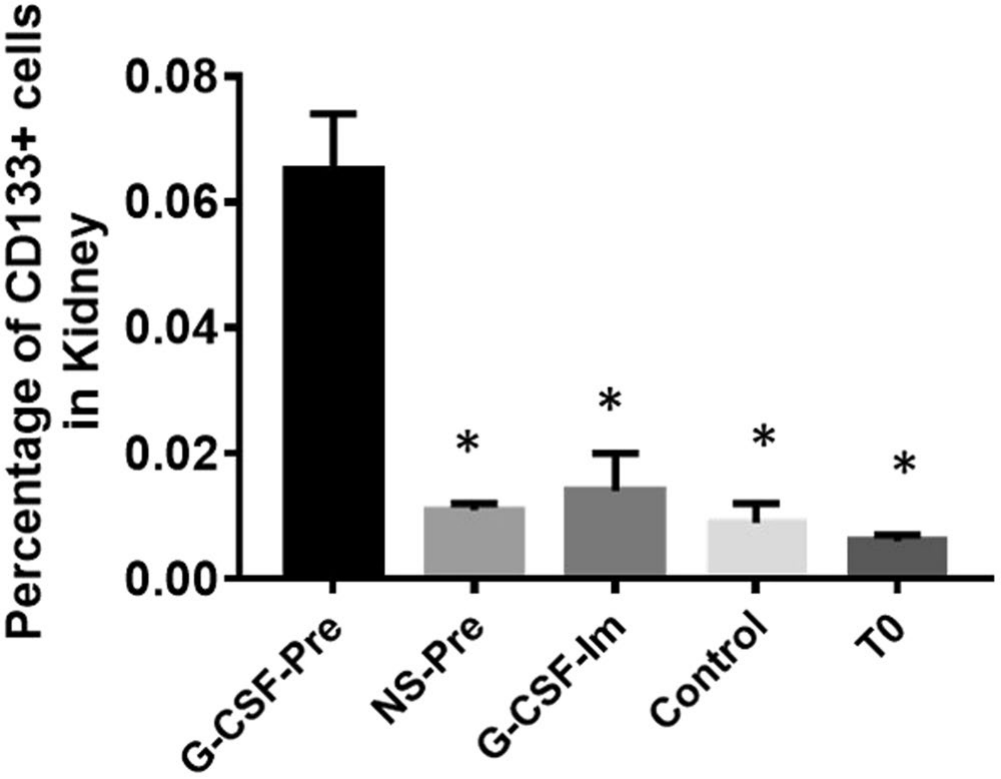
The count of CD133+ cells in mouse G-CSF-Pre group renal tissue is significantly higher than other groups after CPB, G-CSF-Pre group: mice were given G-CSF (10 µg/kg/day) by subcutaneous injection for 2 consecutive days prior to CPB; NS-pre group: mice were subcutaneously injected with normal saline (NS) at 0.5 ml/day for 2 consecutive days prior to CPB; G-CSF-Im group: mice were injected with G-CSF subcutaneously only once, and CPB was performed immediately afterwards; Control group: mice didn’t receive any treatment before surgery; T0 group: mice were treated with G-CSF as above mentioned but no surgery nor CPB performed. *Compared with the G-CSF-Pre group, p < 0.05. (N = 8 of each group).

The experiential mice were divided into 5 groups. G-CSF-Pre group: mice were given G-CSF (10 µg/kg/day) by subcutaneous injection for 2 consecutive days prior to CPB; NS-pre group: mice were subcutaneously injected with normal saline (NS) at 0.5 ml/day for 2 consecutive days prior to CPB; G-CSF-Im group: mice were injected with G-CSF subcutaneously only once, and CPB was performed immediately afterwards; Control group: mice didn’t receive any treatment before surgery; T0 group: mice were treated with G-CSF as above mentioned but no surgery nor CPB performed. Kidney tissue samples were collected as other groups. N = 8 of each group. After CPB, the number of CD133+ cells in renal tissue among groups were measured. As the data presented in Fig. 2, the number of CD133+ cells in T0 group, G-CSF-Pre group, NS-Pre group, G-CSF-lm group and Control group were: 0.006 ± 0.001%, 0.065 ± 0.009%, 0.011 ± 0.001%, 0.014 ± 0.006%, and 0.009 ± 0.003% respectively. Meanwhile, the number of CD133+ cells in the renal tissue of G-CSF-Pre-group mice was significantly greater than that of group NS-Pre, G-CSF-Im, Control and T0 groups. This result proves that G-CSF premobilization stimulates and significantly increase the number of CD133+ cells in the kidney.

### G-CSF premobilization reduced inflammatory factors level in mouse renal tissue

**Figure 3:**
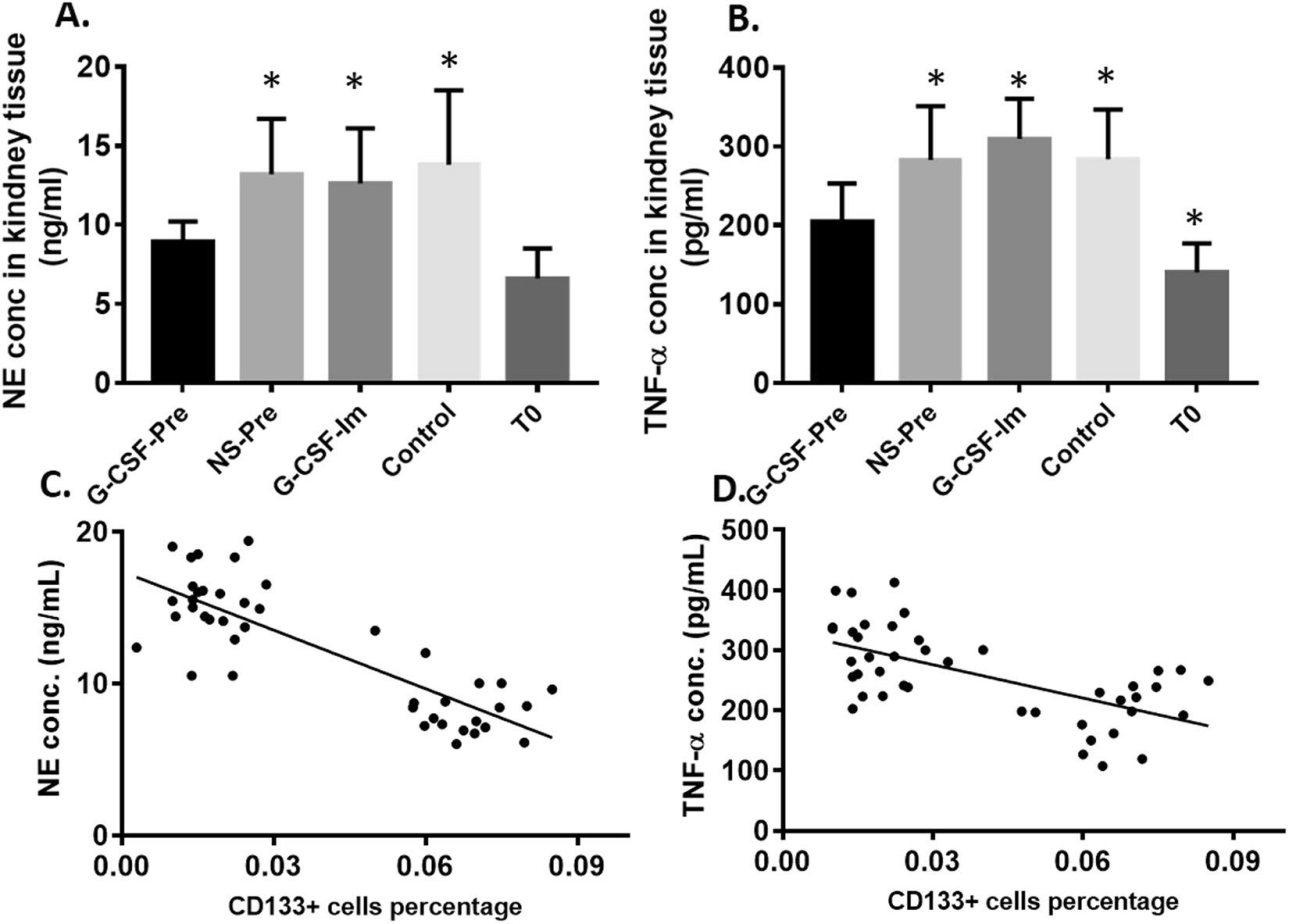
(**A**,**B**) comparison of renal inflammatory factors (TNF-α and NE) in each group. *Compared with the G-CSF-Pre group, p < 0.05. (**C**,**D**) TNF-α and NE have a negative correlation with renal tissue CD133+ cells. *Compared with the G-CSF-Pre group, p < 0.05, there were statistically significant differences.

A recent review and research of the literature on this topic indicated that Neutrophil Elastase (NE) and Tumor necrosis factor alpha (TNF-α) have identified as two of the index markers for tissue inflammatory^44–50^. In order to check the inflammation status after CPB, NE and TNF-α were measured in our experimental mouse system. The Fig. 3A,B indicated that the level of NE and TNF-α level of T0 group was significantly lower than that in the other four groups. This result demonstrated that CPB surgery only has the ability to promote the inflammatory response in the kidney tissue, Meanwhile, the level of NE in G-CSF-Pre-group was significantly lower than that of NS-Pre, G- CSF-Im and Control groups (8.9 ± 1.3 vs. 13.2 ± 3.5 vs. 12.6 ± 3.5 vs. 13.8 ± 4.7 ng/ml, p < 0.05 for each group). The TNF-α concentration in renal tissue also detected by using the Elisa method, after CPB in groups T0 vs. G-CSF-Pre; vs NS-Pre; vs G-CSF-Im; and vs Control shown as: 140.2 ± 36.9 pg/ml; vs 204.4 ± 48.9 pg/ml; vs 282.8 ± 68.5 pg/ml; vs 309.7 ± 50.7 pg/ml; and vs 283.9 ± 62.9 pg/ml respectively, p < 0.05. This highlighted that G-CSF premobilization significantly reduce the level of renal tissue inflammation. Furthermore, a significant negative correlation was observed between NE and CD133+ cells in mouse renal tissue (r = −122.05; P < 0.05) (Fig. 3C), as well as between TNF-α and CD133+ cells in mouse renal tissue (r = −1514.3; P < 0.05) (Fig. 3D),

### Renal tissue pathological staining and injury scores in each group of mice

**Figure 4:**
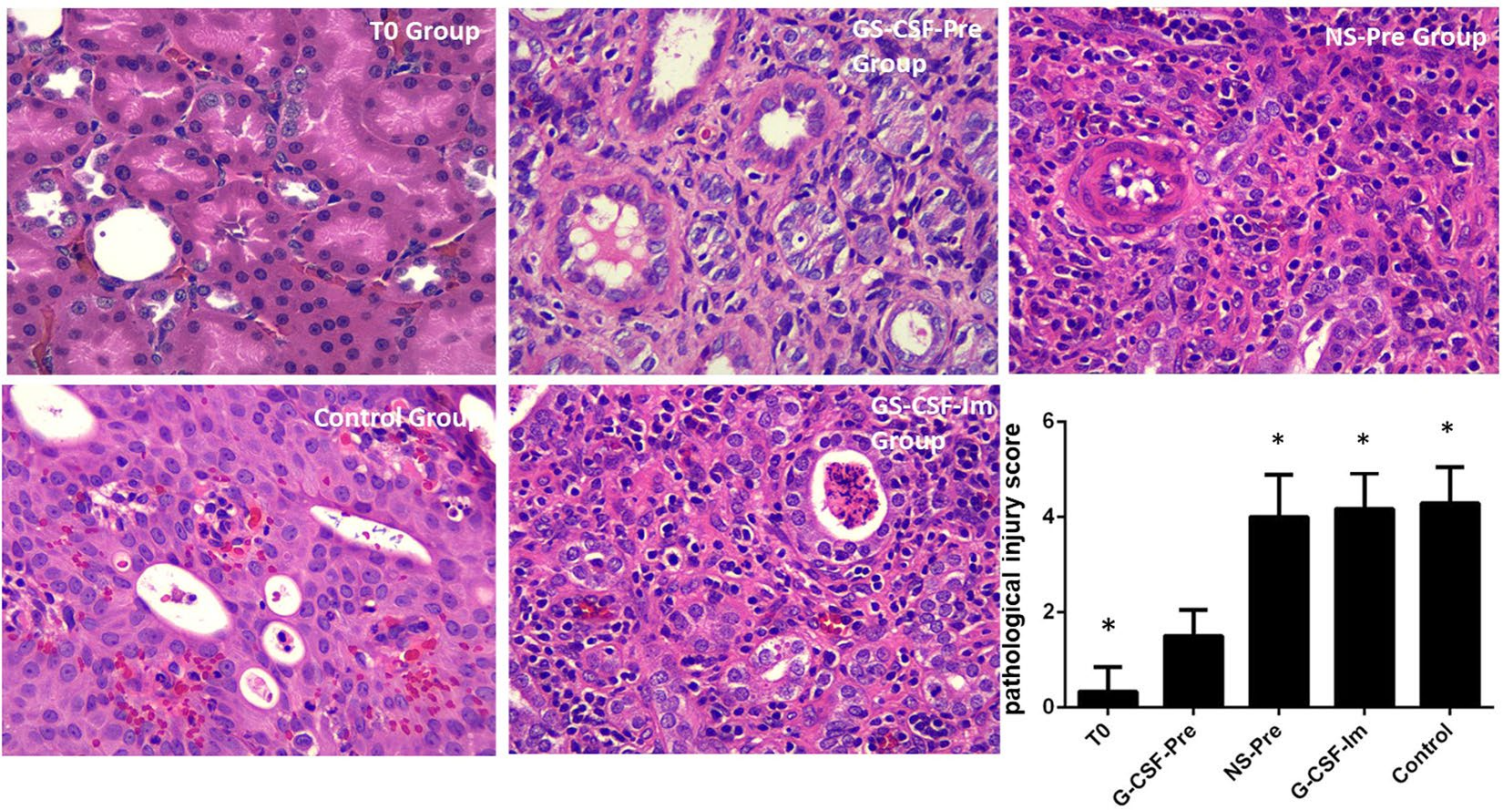
The renal injury induced by IAKI in the G-CSF-Pre-group were significantly mild compared to the other groups. The renal tissue pathological injury score in groups G-CSF-Pre were markedly lower than that in NS-Pre; G-CSF-Im; and Control groups, p < 0.05. *Compared with G-CSF-Pre group p < 0.05.

Renal tissue pathological staining and injury score were used to evaluate renal injury in our study. The apparent edema of HE-stained renal tissue epithelial cells appeared in NS-Pre, G-CSF-Im and Control group, the other indicator of injuries has been found as well, such as bleeding and the signs of tubular injury. Interestingly, these characteristic signs of renal injury in the G-CSF-Pre-group were significantly mild compared to the group listed (Fig. 4). While shown in the T0 control group, the kidney tissue was present in normal and no damage been found. To further quantify the degree of renal damage in each group of mice, we evaluated the pathological grading of renal pathological sections, and the scoring method as described above. The renal tissue pathological injury score in groups G-CSF-Pre were significantly lower than that in NS-Pre; G-CSF-Im; and Control groups, represented as 1.50 ± 0.55; vs 4.00 ± 0.89; vs 4.17 ± 0.75; and vs 4.29 ± 0.76 respectively, p < 0.05. This result indicating that G-CSF pre-treatment provided an efficient way to protect renal tissue from CPB induced IAKI.

### G-CSF premobilization recruit peripheral blood CD133+ cells in renal tissue

**Figure 5:**
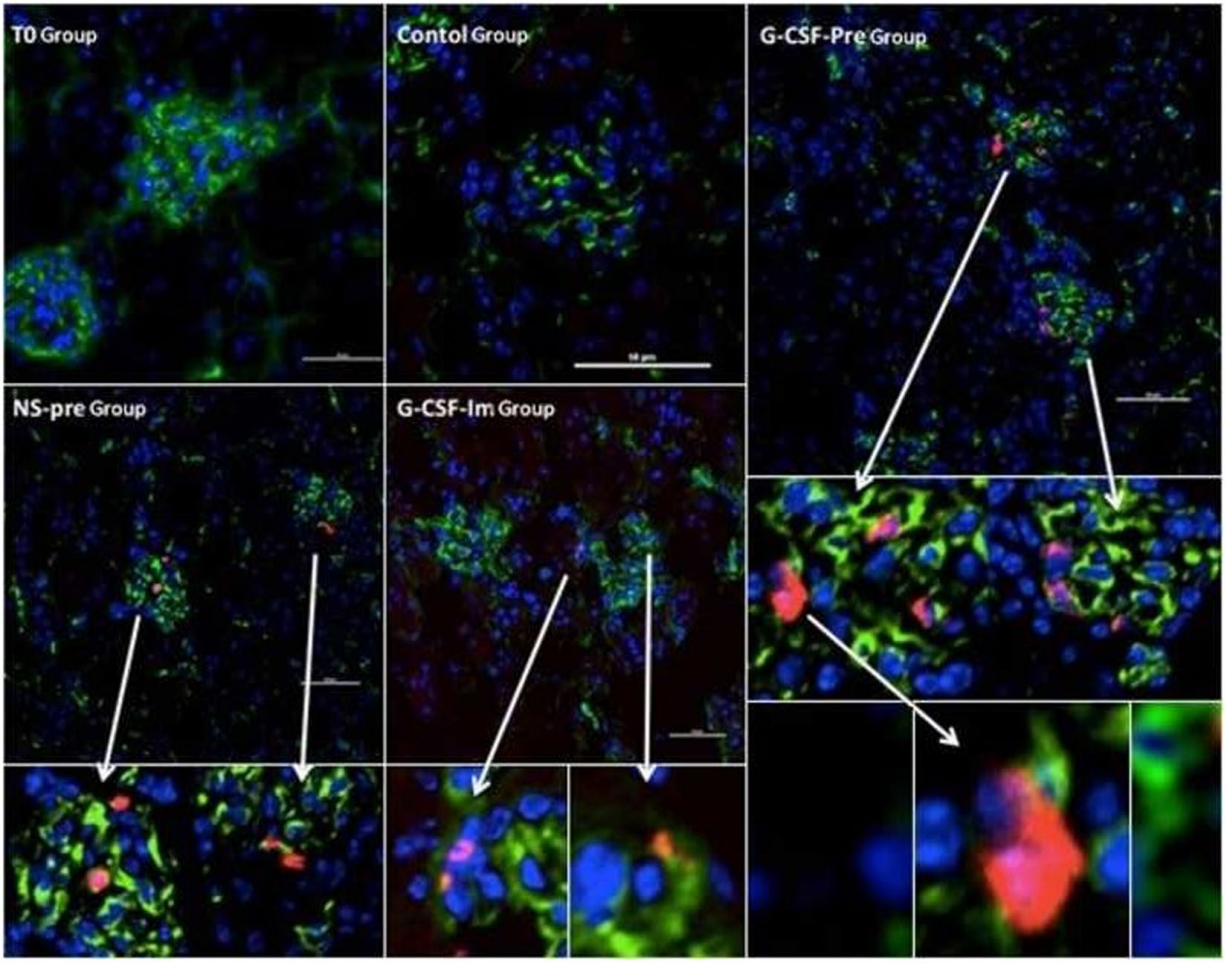
Immunofluorescence staining shows G-CSF premobilization recruit peripheral blood CD133+ cells in renal tissue, CD133 positive staining were shown in red fluorescence color, CD31 was used as the endothelial progenitor cells control displayed in green fluorescence color. CD133+ cells were seen surrounding the glomeruli, G-CSF premobilization recruit more peripheral blood CD133+ cells in renal tissue compared to other experimental groups.

Immunofluorescence staining was employed to locate the CD133+ cells in renal sections; it would evaluate CD133+ cells migration function to the specific injury site. The CD133 positive staining was shown in red fluorescence color, CD31 was used as the endothelial progenitor cells control displayed in green fluorescence color. When CD133+ cells differentiate into endothelial cells, the cells will express both CD133 and CD31 markers, which should be present in the dual color (red with green). As shown in Fig. 5, while CD133+ cells were seen surrounding the glomeruli, G-CSF-Pre group showed more positive CD133 staining, revealed that CD133+ cells migrated to the damaged glomeruli and G-CSF premobilization recruit more peripheral blood CD133+ cells in renal tissue to compare to other experimental groups.

### Renal function has been protected by increasing CD133+ cell numbers in renal tissue by using G-CSF

**Figure 6:**
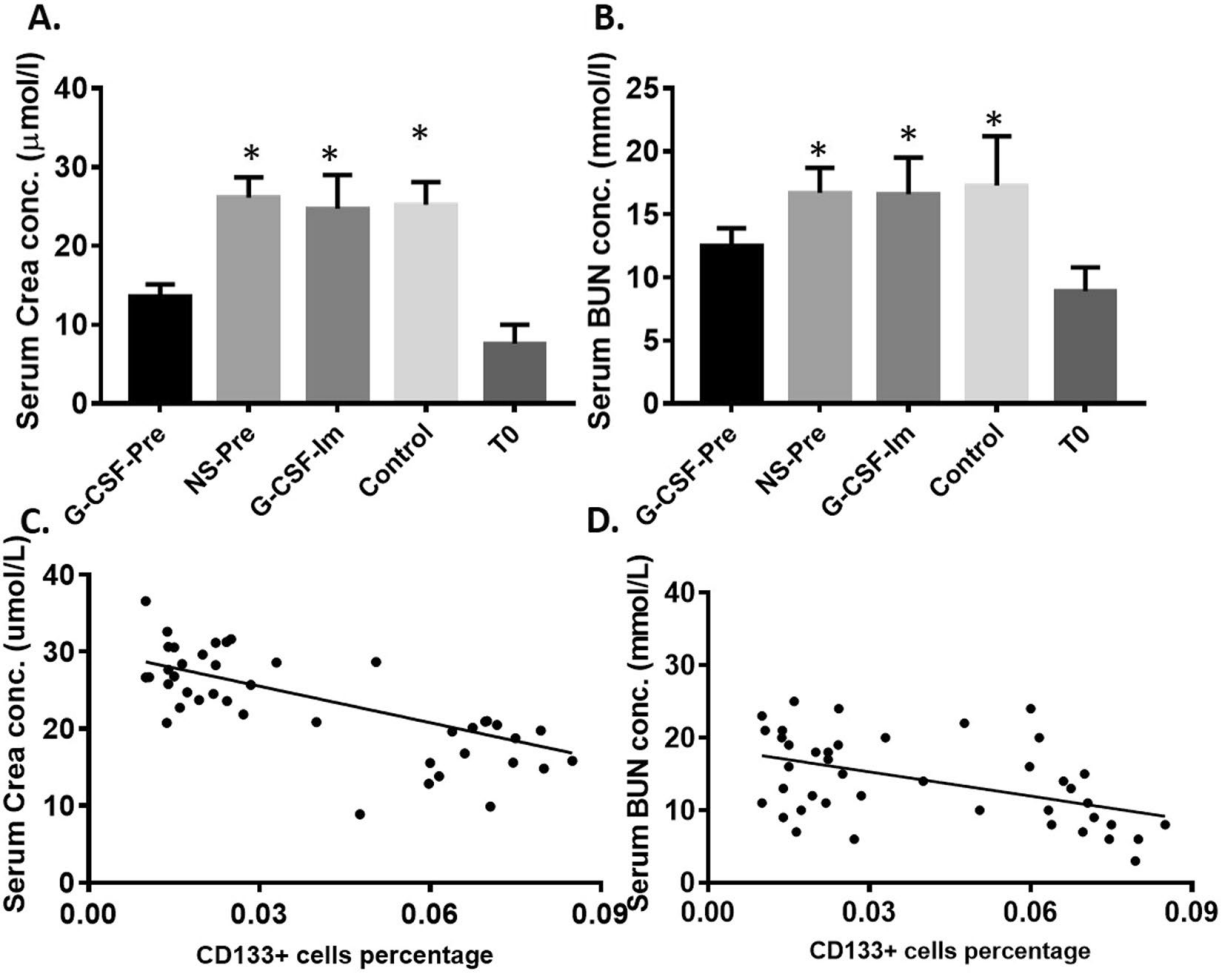
(**A**,**B**) The level of Crea and BUN in G-CSF-Pre group was lower than that of NS-Pre, G-CSF-Im and Control groups. *Compared with the G-CSF-Pre group, p < 0.05, there were statistically significant differences. (**C**,**D**) serum Crea and BUN level have a negative correlation with renal tissue CD133+ cells.

Our previous data have shown that pre-treatment of mice by G-CSF abated renal inflammation after CPB. Then, we measured creatinine levels to see if the renal function was also rescued. In each group, Crea levels were 7.6 ± 2.4 µmol/L vs. 13.5 ± 1.6 µmol/L; vs. 26.1 ± 2.6 µmol/L; vs. 24.7 ± 4.3 µmol/L; and vs. 25.2 ± 2.9 µmol/L for T0 vs. G-CSF-Pre; vs. NS-Pre; vs. G-CSF-Im; and vs. Control, respectively. As shown in Fig. 6A,B, compared to control groups, G-CSF group of mice showed less increased crea and BUN, indicating that the administration of G-CSF will protect renal tissue from IAKI induced injury; Comparison of blood BUN levels between each groups: After CPB the level of BUN in groups T0 vs G-CSF-Pre; vs NS-Pre; vs G-CSF-Im; and vs Control showed as 8.9 ± 1.9 mmol/L vs. 12.5 ± 1.4 mmol/L; vs 16.6 ± 2.9 mmol/L; vs 16.6 ± 2.9 mmol/L; and vs 17.3 ± 3.9 mmol/L respectively, p < 0.05. The renal function results indicated that when premobilization were performed, mice have decreased IAKI related injury. In addition, the correlation was performed and a negative correlation was observed between serum Crea and CD133+ cells in mouse renal tissue (r = −200.23; P < 0.05) (Fig. 6C); between serum BUN and CD133+ cells in mouse renal tissue (r = −83.713; P < 0.05) (Fig. 6D).

## Discussion

Ischemic Acute kidney injury (IAKI) is a rapid deterioration of kidney function and is a common complication after CPB, leading to high mortality and morbidity rates. Unfortunately, there is no effective prevention methods nor treatments currently. Through this mouse model of IAKI induced by CPB, we demonstrated that pretreatment with G-CSF could significantly abate IAKI by increasing the number of CD133+ cells in kidney and circulation. This premobilization also correlated with better renal function after CPB. Our study also gives evidence to the notion that cell therapy could be used as not only a preventive approach, but also provides a potential therapeutic strategy during IAKI occurs. It would be considered as a more useful for treatment of injury because it promotes native repair and regeneration of the body.

Regarding the cells, no specific marker has yet been identified. The frequency of quiescent stage of CD133+ cells in total aspirated human bone marrow mononuclear cells was around 1–2%^51^, however, it has been wildly reported that the CD133+ positive cell in peripheral blood maintains cells proliferation and regenerative potentials^52,53^. Accumulation of peripheral blood CD133+ cells in the renal tissue are likely to promote kidney regeneration. Moreover, in the rodent studies, CD133 ‘progenitor’ cells reported may not exist in the mouse kidney nor in the bone marrow, whether the therapeutic effects of the cells are due to autocrine, paracrine or endocrine effects still remain controversial^23,54–57^. Our results showed that premobilization of CD133+ cells prior to CPB significantly abated IAKI while mobilization of CD133+ cells after CPB procedure couldn’t prevent IAKI efficiently. Previous studies with G-CSF in both animal and human studies also demonstrated similar results that numbers of circulating CD133+ cells only increased 4 hours after the tissue injury^58–60^. Dr. Bi and collaborators described that the joint use of SCF and G-CSF increased the amount of BMSCs in experimental animal kidney tissue and reduced the tissue damage. Increased kidney tissue HIF1α and its target gene products VEGF and EPO expression possibly induce SCF and G-CSF to promote acute tubular necrosis repair^61^. It has been reported that the mobilization of BMSCs by G-CSF has ability to serve as the new strategy for preventing the acute renal injury and rescues the mice from chemotherapy-induced renal failure^24^. All these evidences suggest the necessity and the importance of premobilization to prevent IAKI induced by CPB.

In this study, pre-treatment with G-CSF for 2 days was sufficient to elevate the number of CD133+ in the peripheral and the kidney within 1 hour after CPB. This result suggests that the premobilized CD133+ cells can rapidly home the damaged tissue. Our work also indicated that CD133+ cells possess significant anti-inflammatory ability in both the peripheral and organ, such as kidney. It has been reported that CD133+ cells acted as an anti-inflammation reagent in acute myocardial infarction by Schömig’s group^62^. It is possible that CD133+ cells work in the same way in our system to execute its anti-inflammation function, which supports the paracrine effective function. It could also explain why we observed a subsequent increase in leukocyte count after pretreatment with G-CSF.

In this study, we found that G-CSF pre-mobilized CD133+ cells migrated to the renal tissue relatively early after CPB. We further detected by immunofluorescence staining that the presenting of CD133+ cells mainly focused at the area surrounding the glomerular parts and that no co-expression of CD31 was found. This result indicates that CD133+ cells presented in the renal tissue did not transform into endothelial cells. Further confirmed that CD133+ cells in the early AKI achieve renal protection more through the paracrine mechanism.

Our study opens many promising lines of investigation that may lead to new preventive and therapeutic approaches to CPB-induced IAKI. However, IAKI could be a complication of different surgical procedures such as organ transplantation. Future work should be done on more and different IAKI models to broaden the application of our premobilization method to prevent and treat IAKI. In addition, investigation of the underlining molecular mechanisms should also be addressed to improve this promising method’s efficiency.

## Conclusions

Premobilization of CD133+ cells abated cardiopulmonary bypass-induced ischemic acute kidney injury, by promoting both repairing functions of the damaged epithelium and by its anti-inflammatory activity. G-CSF premobilization stimulates the presenting of CD133+ cells to kidney and significantly increases the number of CD133+ cells, therefore significantly reduces the level of renal tissue inflammation and dramatically improves the renal function after CPB.

These findings stress the remarkable applications of cells-based therapies for potential preventing and treating ischemic acute kidney injury. This study opens up many promising lines of investigation that may lead to new preventive and therapeutic approaches to CPB-induced IAKI. Further studies are needed to yield encouraging and promising results in the field of cells as therapeutic agents for kidney injury.

### Ethics approval and consent to participate

All animals received standard care according to the Guide for the Care and Use of Laboratory Animals of the US National Institutes of Health. This study was approved by the Sichuan University Ethics Committee and registered with the China Clinical Laboratory Registration Center (No. ChiCTR- OCS-09000398; www.chictr.org/).

## Data Availability

The datasets used and/or analyzed during the current study available from the corresponding author on reasonable request.
